# Design Techniques to Optimize the Scaffold Performance: Freeze-dried Bone Custom-made Allografts for Maxillary Alveolar Horizontal Ridge Augmentation

**DOI:** 10.3390/ma13061393

**Published:** 2020-03-19

**Authors:** Felice Roberto Grassi, Roberta Grassi, Leonardo Vivarelli, Dante Dallari, Marco Govoni, Gianna Maria Nardi, Zamira Kalemaj, Andrea Ballini

**Affiliations:** 1 Department of Basic Medical Sciences, Neurosciences and Sense Organs, University of Bari “Aldo Moro”, 70121 Bari, Italy; 2 Department of Surgical, Medical and Sperimental Sciences, University of Sassari, 07100 Sassari, Italy; grassi.roberta93@gmail.com; 3 Reconstructive Orthopaedic Surgery and Innovative Techniques—Musculoskeletal Tissue Bank, IRCCS Istituto Ortopedico Rizzoli, 40124 Bologna, Italy; leonardo.vivarelli@ior.it (L.V.); dante.dallari@ior.it (D.D.); marco.govoni@ior.it (M.G.); 4 Department of Oral and Maxillo-Facial Sciences, University of Rome "La Sapienza", 00148 Rome, Italy; giannamaria.nardi@uniroma1.it; 5 Private Practice, 20139 Milano, Italy; zamirakalemaj@hotmail.com; 6 Department of Biosciences, Biotechnologies and Biopharmaceutics, Campus Universitario "Ernesto Quagliariello", University of Bari "Aldo Moro", 70121 Bari, Italy; 7 Department of Precision Medicine, University of Campania "Luigi Vanvitelli", 80123 Naples, Italy

**Keywords:** geometry optimization of scaffolds, allograft, block bone grafts, custom made bone, design techniques for scaffold, precision and translational medicine

## Abstract

The purpose of the current investigation was to evaluate the clinical success of horizontal ridge augmentation in severely atrophic maxilla (Cawood and Howell class IV) using freeze-dried custom made bone harvested from the tibial hemiplateau of cadaver donors, and to analyze the marginal bone level gain prior to dental implant placement at nine months subsequent to bone grafting and before prosthetic rehabilitation. A 52-year-old woman received custom made bone grafts. The patient underwent CT scans two weeks prior and nine months after surgery for graft volume and density analysis. The clinical and radiographic bone observations showed a very low rate of resorption after bone graft and implant placement. The custom-made allograft material was a highly effective modality for restoring the alveolar horizontal ridge, resulting in a reduction of the need to obtain autogenous bone from a secondary site with predictable procedure. Further studies are needed to investigate its behavior at longer time periods.

## 1. Introduction

Implant-supported rehabilitation of the edentulous ridge requires adequate volume and integrity of the alveolar bone [1].

Bone resorption in the maxillary ridge, due to trauma, pathology or tooth loss, frequently results in a knife-edged deformity, which complicates implant placement and stabilization, particularly in the posterior jaw [2,3,4]. Grafting with allograft bone has been documented to be a useful tool in reconstructing jaw anatomy, [5] restoring esthetics [6], and providing biomechanical support for the placement of dental implants [7].

Clinically, the most suitable banked bone allografts are fresh-frozen (FFBAs), freeze-dried bone allografts (FDBAs), and demineralized freeze-dried (DFDBAs) [8], although in oro-maxillofacial surgical interventions FDBAs and DFDBAs are the most used.

Frozen bone is accessible for human receivers after at least 6 months of quarantine at −80 °C [9] and no additional preparation is required. Moreover, the osteoinductive proteins are preserved [10].

Strict guidelines for tissue harvesting and storing at −80 °C make the risk of primary infections and antigenicity reasonably low [11]. In addition, the reduction of water content in frozen bone by the freeze-drying process further decreases potential microbial contaminations [12].

Frozen bone and relative freeze-dried/demineralized products are accessible as cancellous granules/blocks, corticocancellous granules/blocks, and cortical granules or chips. Once defrosted, frozen bone has handling qualities similar to fresh bone [8] while freeze-dried allografts have the additional advantage of storage at room temperature.

Bone density can be measured with high reproducibility by means of Cone-Beam-Computed-Tomography (CBCT) scans [13]. Further techniques, such as orthopantomography, do not assure fitting precision in density determination [13].

The aim of the present translational study was to evaluate the clinical success of horizontal ridge augmentation in severely atrophic maxilla (Cawood and Howell class IV) using custom made bone harvested from the tibial hemiplateau of cadaver donors.

## 2. Materials and Methods

A 52-year-old woman presented compromised anterior maxillary ridges, who presented for the placement of dental implants, was included in this pilot study. Written and verbal information was given to the patient before enrollment, and written informed consent was obtained. The study was conducted in full accordance with the World Medical Association Declaration of Helsinki on experimentation involving human subjects, as revised in 2008.

Inclusion criteria was a horizontal severely atrophic maxilla (Cawood and Howell class IV), needing a bone grafting procedure prior to implant placement. Exclusion criteria were established according to Venet et al. (2017) [12]. Plaque index score was maintained ≤25% throughout the study [14].

### Graft Sample Blocks

The processing was performed on corticocancellous bone blocks obtained from a proximal tibial epiphysis, in the anatomical region between the articular surface of the tibial plateau and tuberosity.

Human allogeneic bone blocks were collected from cadaveric donors, stored at −80 °C and processed in the accredited public non-profit Musculoskeletal Tissue Bank of IRCCS Istituto Ortopedico Rizzoli (Bologna, Italy), authorized by the Italian National Transplant Center for the collection, processing, and distribution of human musculoskeletal tissue [15], and registered in the European Tissue Establishment list (code IT000096). The choice of the block to be machined involved considering dimensions slightly greater than the machining area defined with the graft design.

After thawing, each block was fixed with clamps on a special stainless-steel table in a GMP-Class A (Good Manufacturing Practice) Clean Room environment. Then, the table was fixed inside the CNC (Computer Numerical Control) milling machine (model Bright, Delta Macchine, Rieti, Italy). After tool fixing, a 3 mm diameter ball mill, the execution of the machining trajectory was started and accurately monitored by the operator.

At the end of the processing, the block was removed and finished, the supports were broken by hand and sharp edges, and rough corners were hand-refined using sterile rasps. Then, grafts were cleaned with organic solvents, washed with sterile water, and freeze-dried (VirTis Genesis 25, SP Scientific, Warminster, PA, USA). Finally, grafts were individually wrapped in triple pack. Microbiological sampling was performed during the processing of grafts and after the lyophilization protocol in order to exclude microbial contamination and declare tissues suitable for implantation.

According to the Guidelines of the Italian National Transplant Center, lyophilized bone grafts are preserved at room temperature for a maximum of 5 years.

### Graft Design

**Figure 1:**
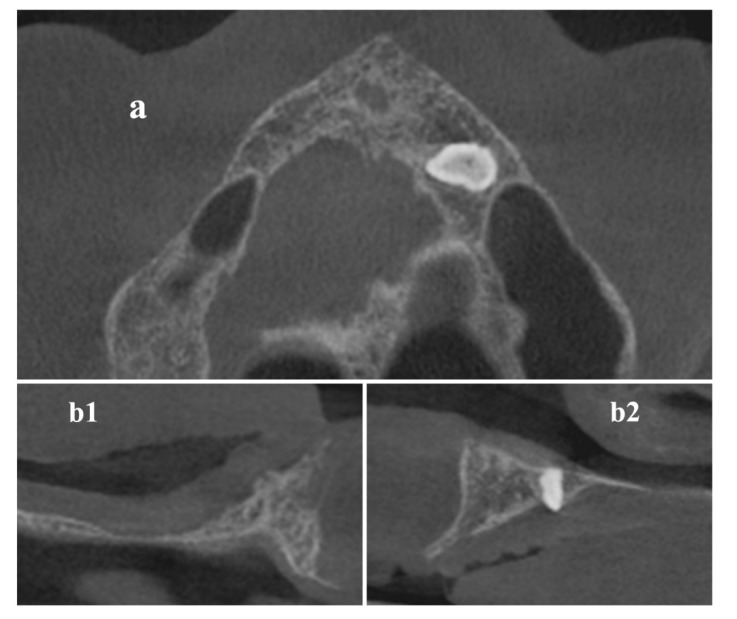
Cone-Beam-Computed-Tomography (CBCT) axial (**a**) and cross-sectional (**b1,b2**) images before surgical procedure.

Digital Imaging and Communications in Medicine (DICOM) data of the maxilla (Figure 1) were acquired by a Cone Beam Computerized Tomography (CBCT) scanner (Orthophos XG 3D Ceph, Dentsply Sirona Italia Srl, Roma, Italy) and imported into the 3DSlicer software (www.slicer.org) [16,17,18].

After setting a threshold for the automatic selection of the areas delimiting the cortical bone, a manual analysis for each slice was performed to correct potential errors. Starting from the selected areas, an automatic procedure generated the 3D model as a stereolithography (STL) file.

The graft design was performed by Rhinoceros ver. 4 (www.rhino3d.com) following these steps: (a) placement of a parallelepiped in the position where the volume increase was required; (b) Boolean subtraction between the parallelepiped and the STL model of the patient’s anatomy; (c) revision of the model for the manufacture by a 3-axis milling machine to obtain a L-shape section. This procedure was performed for each graft.

**Figure 2:**
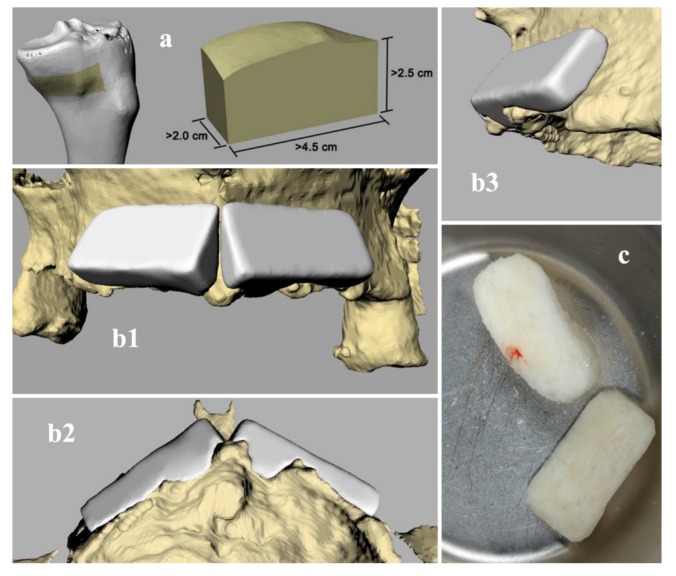
(**a**) Render of 3D bone donor site harvested from a cadaver tibial hemiplateau. (**b1**–**b3**) Render of 3D reconstruction of patient's anatomy (yellow) and designed bone grafts (white). (**c**) Final block for clinical use.

The design of the grafts has been repeatedly validated and subsequently corrected following the surgeon’s indications, up to the final model (Figure 2).

### Trajectory Planning and Graft Manufacturing

The planning of machining trajectories was performed using Rhinoceros by the Rhino-CAM 2 plug-in (MecSoft Corporation, Irvine, CA, USA). Each graft was positioned in the center of the machining CAM area with the larger flat area facing down to keep the cortical portion of the tissue intact, in order to enhance the graft resistance during processing and avoid breaking during implantation. Bone bridges have been added to the CAD design to maintain fixation during processing and removed by hand at the end of the graft manufacturing procedure. The working area was defined according to the design’s dimensions and the diameter of the milling tool.

**Figure 3:**
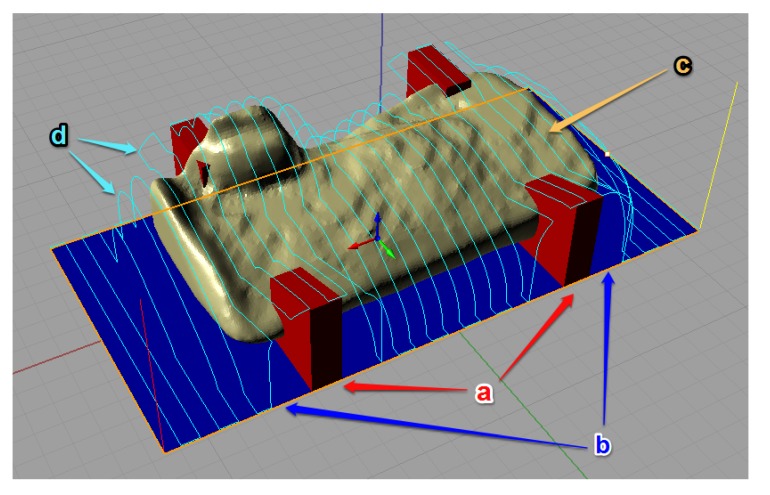
Tool-path trajectory planning: (**a**) added bone bridges; (**b**) work area; (**c**) graft design; (**d**) tool-path.

The tool-path trajectories have been programmed, subsequently simulated and modified to obtain the best result (Figure 3). Then, the definitive trajectories have been exported as G-Code coding specific for the milling machine used.

The machining operation was composed by two different phases: horizontal roughing and parallel finishing. The tool used for each phase was the same, a fluted 3 mm ball mill with two teeth, hard metal k10 material (tungsten carbide). In each phase rotational velocity of the tool was 5300 RPM and feed rate was 200 mm/min, that corresponds to a feed per tooth of 19 μm and a tangential velocity of 50 m/min (0.833 m/s). Horizontal roughing was performed as a full immersion vertical milling, with a machining depth of 3.5 mm, leaving a stock margin of 0.3 mm. Parallel finishing was a vertical milling, with a machining depth depending on stock margins and parallel steps of 0.7 mm (Figure 3d) leaving a variable stock margin between 0 mm and 0.15 mm (a sufficient dimensional accuracy for oral implant surgery). This machining strategy, performed by few passages, reduced the machining time and the bone heating during the process. Although temperature was not monitored during the fabrication of these two custom grafts, our machining parameters, especially the small feed per tooth, allow a local temperature between 40 °C and 60 °C for cortical bone and lower values for cancellous bone. According to Krause [19], these parameters do not cause the denaturation of proteins and enzymes that occurs for temperature above 70 °C.

As reported by Van Isacker et al. [20], frozen bone can be handled and reshaped like normal bone and is fully workable, but freeze-dried bone, unless rehydrated in saline, is brittle like ceramic, and is not fully workable.

Thus, to execute a better processing procedure and to obtain a further reduction in temperature, machining was performed on a frozen bone block. At the end of the machining operation, the custom allograft was freeze-dried.

### Surgical Procedure

**Figure 4:**
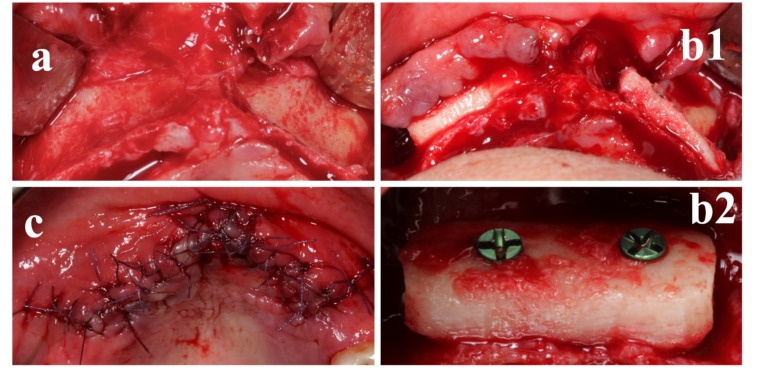
The custom-made freeze-dried bone allograft (FDBA) was positioned in the deficit area and stabilized with a screw. (**a**) Anatomical site prior surgical procedure and clinical view of the defect. (**b1,b2**) Screw positioning on a custom-made FDBA. (**c**) Sutures.

Local anesthesia was obtained by infiltrating articaine (4% containing 1:100,000 adrenaline). The exposure of the three-dimensional aspect of the bone defects was achieved through a full-thickness crestal incision with two vertical releasing incisions (Figure 4a).

The clinically sized, anatomically shaped, custom-made bone block was placed in position strictly overlapping the underlying alveolar crest and fitted securely to the residual bone (Figure 4b1). The recipient site was weakened with multiple micro-holes to enhance bleeding from the trabecular bone [12,21,22]. Rigid fixation of the scaffold to the residual crest was obtained by means of a titanium mini-screw (1.5 mm width, 8 mm length) (Tekka by Global-D, Lyon, France) (Figure 4b2) [12].

The grafted area was closed with a pulley suture for proper flap adaptation and to avoid any tissue strangulation by an absorbable 4.0/5.0 suture material. Sutures were removed 14 days postoperatively (Figure 4c) and no prosthetic device was admitted in the following 90 days, with particular care in domiciliary oral hygiene procedures.

**Figure 5:**
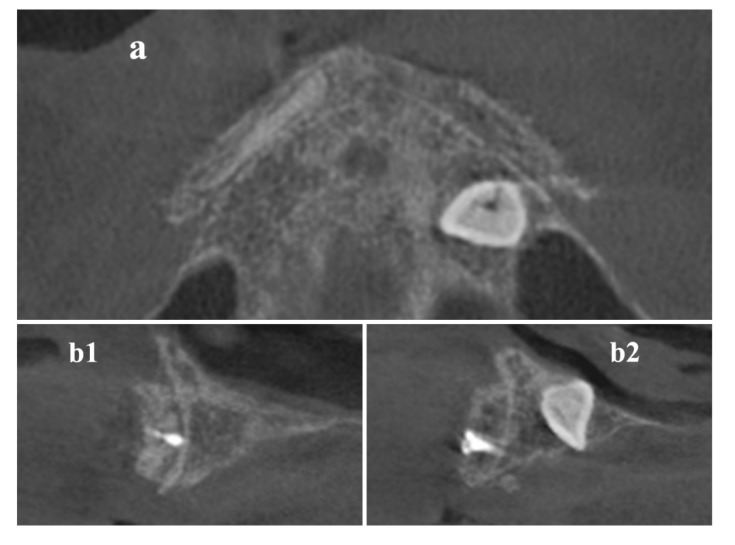
CBCT scans were done after 9 months to define implant placement width and length, on (**a**) axial and (**b1,b2**) cross-sectional images.

Preceding to the second phase, supplementary CBCT scans were performed to evaluate grafts gain (Figure 5).

**Figure 6:**
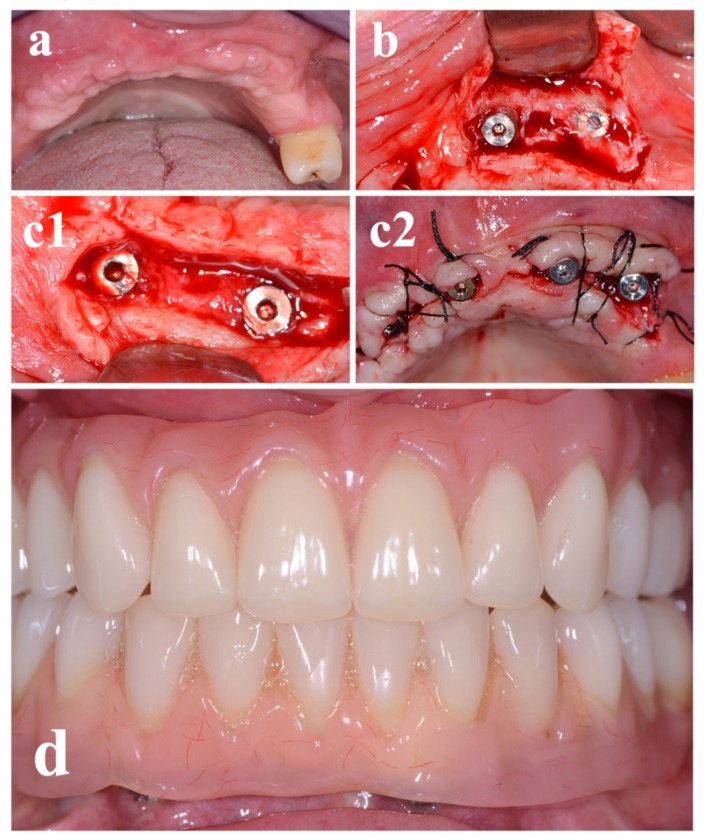
Nine months after the surgical procedure, the implant was positioned. (**a**) Soft tissues before surgical procedure. (**b**) Surgical flap is elevated. (**c1**) Implant was positioned and (**c2**) sutures performed. (**d**) Frontal view of the final rehabilitation.

After a 9 month healing period, micro-screws were removed (Figure 6a,b), and 3.7 mm in diameter for a 10 mm in length dental implants (iRES SAGL, Mendrisio, Switzerland) were placed, and sutures performed (Figure 6c). No supplementary grafting procedure was required in the current investigation [22].

### Statistical Evaluation

The outcome values were analyzed using the *t-test* for paired samples for pre–post differences with time as the factor and IBM Statistical Package for the Social Sciences (SPSS Inc. Version 21.0, Chicago, IL, USA) software to detect significant differences between pre-test and post-test scores obtained from bone volume analysis and measurements of variation in ridge thickness from CBCT images at 9 months after allograft insertion.

## 3. Results

A patient presenting atrophic anterior maxillae was selected to participate in the study. The patient received 2 custom-made allografts. The healing period was uneventful.

At the crestal level, the patient met the inclusion criteria of ridge width around 2 mm [22]. The titanium fixation mini-screws detached and bleeding from the bone graft were detected, demonstrating revascularization of the site.

Moreover, the regenerative surgical procedure went well. In fact, the custom-made allograft scaffolds perfectly fitted in the bone anatomy and were therefore easily adapted to the bone defects during surgery, secured by titanium mini-screws (Figure 4, Figure 5 and Figure 6). This excellent matching of the size/shape helped the surgeon to reduce the operation time [23]. Moreover, all implants were inserted with fitting primary stability (Figure 6d).

Paired comparisons showed a significant mean increase in ridge thickness of 5.0 ± 0.55 mm from the preoperative measurement to the postoperative measurement (p < 0.01).

The bone resorption that occurred during the incorporation period corresponds to 7.8% (95% confidence interval (CI): (6.4%, 9.2%)) of the measured postoperative ridge thickness.

## 4. Discussion

Maxillary ridge augmentation relates to procedures designed to correct a thin alveolar ridge. For dental implant placement, adequate bone volume is necessary, unfortunately, bone volume is not always adequate, particularly in elderly patients.

Through the current improvements in planning and manufacturing software and hardware, graft customization can be combined with the implant dentistry digital workflow with the potential patients to become part of daily clinical practice [24].

The reduction of risk factors such as infections caused by contamination of surgical site, or contamination of the graft during preoperative preparation, can be obtained with the manufacturing of a custom sterile graft [23]. The correct design of a custom graft, with also a reduction of surgical time, can be obtained with a proper planning of surgical procedure.

Nevertheless, the sterile manufacturing of bone in a GMP-Class A environment, without the need of terminal sterilization via gamma radiation, guarantees the safety of graft in terms of sterility and the quality of tissue in terms of biological properties [25,26].

Among bone replacement grafts, autograft is considered the gold standard [27]. However, owing to the low level of patient acceptance of autografts, allografts (grafts from the same species) have become the most popular bone grafts in oral surgery. Among allografts available for the periodontal regeneration, freeze-dried bone allograft (FDBA) and the demineralized freeze-dried allograft (DFDBA) are the allograft-forms most used [28].

Although FDBAs and DFDBAs were first used in periodontal therapy in early 1970s, to date they continue to be the most requested allografts in clinical practice because of their osteoconductive and osteoinductive properties, respectively [29]. Compared to autogenous bone, allografts feature several advantages such as adequate availability and the elimination of additional donor site surgery and morbidity [30]. Besides, the freeze-drying technique removes more than 95% of water content from bone, allowing further storage of tissues at room temperature and reducing antigenicity [31]. In addition, the application of stringent tissue banking guidelines together with bone processing and liophilization protocols performed in a GMP-Class A environment, minimize the risk of microbial contaminations [32], and reduce the need for terminal sterilization by gamma rays and consequent loss of mechanical strength of bone tissues [33]. As a matter of fact, although in freeze-dried bone tissues most of the water content has been removed, ionization has a direct effect on the collagen fiber bundles causing the breakage of protein chains and hence alters mechanical resistance [34].

Since only the demineralized bone matrix retains substantial amounts of endogenous osteoinductive proteins such as bone morphogenetic proteins (BMPs), known to promote bone growth and regeneration (i.e., BMP-2, BMP-4, BMP-7) [35,36], FDBAs are considered only as osteoconductive allografts. However, their structure can serve as a scaffold and support for the host cells that can migrate into the grafts and then differentiate into new osteogenic cells. In addition, in respect to DFDBAs, the superior mechanical properties of FDBAs make these allografts more suitable for restoring the alveolar horizontal ridge in athrophic maxillae.

Hence, the findings of our study show, with a high success rate, that FDBAs represent a reliable treatment option for extensive rehabilitation of atrophic maxillae, consistent with findings reported with the use of autologous bone.

The success rate of the block grafts was very effective and comparable with those reported by other authors [37,38,39]. Besides, the augmentation procedure permitted the insertion of implants in the grafted area 9 months after surgery. The clinical and radiographic observations showed a very low rate of bone resorption of the graft material, improving the ability to place endosseous implants.

A potential scaffold frontier for bone tissue engineering protocols seems to be the mesenchymal stem cells seeded in nanocomposite materials with antibacterial properties, but further studies in this direction are necessary prior to be consider it as a safe and useful tool for human implantation purposes [40].

Our data showed that custom-made FDBA can be used as successful graft material for the treatment of bone maxillary ridge defects. If adequate surgical techniques are adopted, this type of bone graft can be safely used in regions of implant placement as a suitable alternative to autogenous grafts. Furthermore, the personalized design of custom grafts and sterile machining in a GMP-Class A environment, as described above, can be applied to different surgical specialties, such as orthopedics, spinal surgery, and maxillofacial surgery.

## 5. Conclusions

This pilot study demonstrated the possibility of fabricating customized CAD/CAM grafts from the tibial hemiplateau of cadaver donors. The grafts were digitally designed based on CBCT scans of partially dentate patients using a set of 3D software, showing that grafts dimensions were correlated to the defect type. The sterile manufacturing of grafts resulted in the correct reproduction of the designed graft, leading to a correct matching with the bone surface of patient. Additional *in vivo* studies with custom-made bone allografts are required for the validation of this digital workflow for maxillary bone augmentation.
